# Primary jejunal gastrinoma: a case report and review of the literature

**DOI:** 10.1186/s12957-015-0728-x

**Published:** 2015-11-06

**Authors:** Kevin A. Wang, Yenn-Hwei Chou, Shu-Han Huang, Tong-Jong Chen

**Affiliations:** Division of General Surgery, Department of Surgery, Shin Kong Wu Ho-Su Memorial Hospital, No. 95, Wenchang Rd., Shilin Dist., Taipei, 11101 Taiwan Republic of China; Department of Pathology and Laboratory Medicine, Shin Kong Wu Ho-Su Memorial Hospital, No. 95, Wenchang Rd., Shilin Dist., Taipei, 11101 Taiwan Republic of China

**Keywords:** Zollinger-Ellison syndrome, Gastrinoma, Jejunum

## Abstract

**Background:**

Primary jejunal gastrinomas are exceedingly rare, and data for long-term follow-up is limited. Until now, only six cases of gastrinomas arising from the jejunum have been reported in the English literature.

**Case Presentation:**

Presented is a case of a primary gastrinoma located in the proximal jejunum. After surgical resection of the tumor, eugastrinemia was quickly achieved and after a 10-year follow-up period, the patient was still disease-free.

**Conclusions:**

This case report demonstrates that surgical resection of a primary jejunal gastrinoma without evidence of metastasis can be curative, with a good long-term prognosis.

## Background

A gastrinoma is a non-beta islet cell tumor which secretes gastrin. The hypergastrinemic state caused by a gastrinoma results in multiple recurrent and often refractory ulcers in the gastrointestinal tract and is known as Zollinger-Ellison syndrome (ZES). In 1955, ZES was originally reported with the presenting triad of recurrent peptic ulceration, hypersecretion of gastric acid, and the presence of a non-beta islet cell tumor of the pancreas [1]. Since then, it has been well-documented that most primary gastrinomas are found mainly in the duodenum, pancreatic head, or lymph nodes within the traditional gastrinoma triangle which is demarcated by the junction of the cystic duct and common bile duct, junction of the second and third portion of the duodenum, and the junction between the neck and body of the pancreas [2].

However, there have been reported cases of gastrinomas outside of these areas, which have been called ectopic gastrinomas. About 5.6 % of patients have a primary gastrinoma located in an ectopic site [3]. Primary jejunal gastrinomas are exceedingly rare, and data for long-term follow-up is limited. Until now, only six cases of gastrinomas arising from the jejunum have been reported in the English literature [3–7]. Presented is a case of a primary gastrinoma located in the proximal jejunum, which is a possible diagnosis when a jejunum tumor is detected by radiographic examination. Ten years after segmental resection of the jejunum containing the gastrinoma, the patient was still eugastrinemic and asymptomatic, and there was no evidence of tumor recurrence.

## Case presentation

In September 2003, a 34-year-old male patient presented at the Department of Surgery at Shin Kong Wu Ho-Su Memorial Hospital (Taipei, Taiwan) with abdominal fullness and discomfort that had gradually developed over the past year. Upon further review, the patient had an 8-year history of heartburn which was only slightly relieved by antacids. Bowel habits were normal without mention of tarry or bloody stool. Also, he did not notice recent body weight loss.

**Fig. 1:**
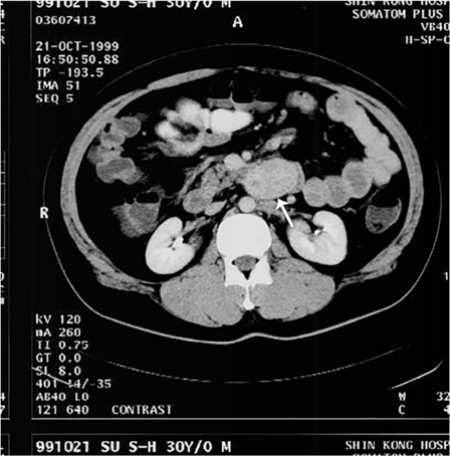
Contrast abdominal computed tomography showing a 7 cm × 6 cm × 4 cm mass in the proximal jejunum (*white arrow*)

**Fig. 2:**
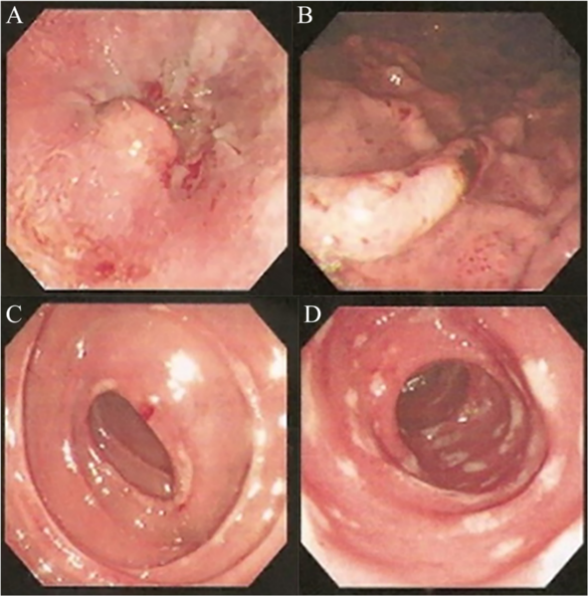
Findings of esophagogastroduodenoscopy and small-bowel endoscopy. **a** Circumferential reflux esophagitis with superficial ulceration was observed in the distal esophagus near the gastroesophageal junction. **b** Gastric ulcers with hemorrhage were noted. **c** Multiple erosions were found in the duodenum. **d** Several upper jejunal ulcers were also noted

Abdominal ultrasound in September of 2003 showed an abdominal mass in the left upper quadrant of his abdomen. An abdominal computed tomography (CT) confirmed a left upper quadrant abdominal mass (approximately 7 cm × 6 cm × 4 cm) near the proximal jejunum (Fig. 1). Esophagogastroduodenoscopy and small-bowel endoscopy revealed several esophageal, gastric, duodenal, and upper jejunal ulcers (Fig. 2). Serum gastrin levels were elevated (451.00 pg/mL; normal range 30–100 pg/mL). Serum tumor markers including alpha-fetoprotein (AFP), carcinoembryonic antigen (CEA), and carbohydrate antigen 19-9 (CA 19-9) were within normal limits. The presence of multiple ulcers, ulcers distal to the first portion of the duodenum, refractory symptoms of gastroesophageal reflux disease, and elevated fasting serum gastrin levels raised the suspicion of Zollinger-Ellison syndrome. Endoscopic ultrasound (EUS) was performed to localize a possible tumor in the gastrinoma triangle but was unable to identify any tumor.

An exploratory laparotomy was performed. At surgery, a large tumor was identified in the wall of the proximal jejunum, approximately 3 cm distal to the ligament of Treitz. Thorough exploration of the abdomen, including the liver, small intestine, pancreas, stomach, duodenum, mesentery, and retroperioneal regions in the upper abdomen and concomitant intraoperative ultrasonography (US) did not reveal any other tumors. Segmental resection of the upper jejunum, including a 7 cm × 6 cm ×4 cm tumor just distal to the ligament of Treitz, was done, and subsequent jejunal-jejunal end-to-end anastomosis was performed.

**Fig. 3:**
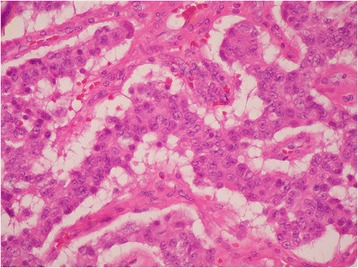
Hematoxylin and eosin stain (×400). The tumor had relatively uniform cells having centrally located nuclei and eosinophilic cytoplasm

**Fig. 4:**
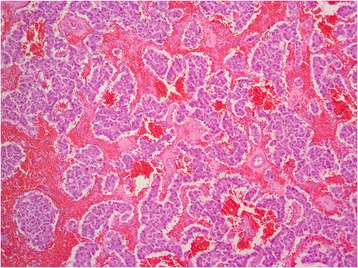
Hematoxylin and eosin stain (×100). The tumor cells were arranged in trabecular and gyriform fashions

**Fig. 5:**
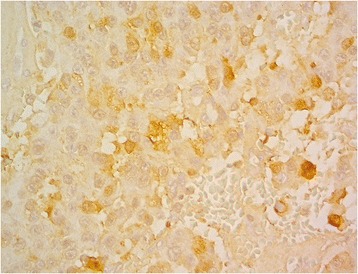
Gastrin stain (×400). Immunohistochemical stains were positive for gastrin

Pathological findings of the jejunal tumor showed a neuroendocrine tumor consistent with gastrinoma. Microscopically, the tumor was composed of relatively uniform cells having centrally located nuclei and eosinophilic cytoplasm (Fig. 3). The tumor cells were arranged in trabecular and gyriform fashions (Fig. 4). The mitotic index was low, and no tumor necrosis was found. On immunohistochemical studies, the tumor cells were positive for gastrin (Fig. 5).

On the fifth postoperative day, his fasting serum gastrin had decreased to 65.8 pg/mL. He was discharged 9 days postoperatively in fair condition without any complications. The patient was regularly followed up, and over 10 years after surgery, the patient remains free of acid regurgitation and abdominal discomfort and is in good health. Current gastrin levels are normal (75.60 pg/mL), esophagogastroduodenoscopy revealed no ulcers, and recent CT and magnetic resonance imaging (MRI) showed no signs of recurrent tumors.

### Discussion

Primary jejunal gastrinomas are exceedingly rare, and data for long-term follow-up is limited. To our knowledge, only six cases of gastrinomas originating from the jejunal wall have been reported in the English literature to date (Table 1) [3–7].

**Table 1:** Reported cases of jejunal gastrinomas including location, surgical procedure, follow-up period, and outcome

Author, year	Sex/age (years)	FSG level (pg/mL)	Location of jejunal gastrinoma	Size (cm)	Procedure	Follow-up	Outcome
Norton et al. 1986	M/66	5650	Around the ligament of Treitz	3	Tumor excision	6 months	No recurrence
	F/35	460	Around the ligament of Treitz	0.5	Tumor excision	3 months	Abnormal FSG at 3 months
Antonioli et al. 1987	F/46	730–980	5 nodules in the jejunal wall, 10 cm distal to the ligament of Treitz, with adjacent mesenteric lymph node metastasis	0.4–3	Partial resection of involved jejunum and mesentery	42 months	Alive and remained eugastrinemic
Katoh et al. 1990 [6]	M/25	764	A primary gastrinoma in the jejunal wall just distal to the ligament of Treitz. Two more tumors in adjacent jejunal mesentery. Multiple liver metastases	NP	Excision of jejunum tumors + remnant gastrectomy	10 years	Alive and in stable condition
Nishiwaki et al. 1992 [7]	M/63	787–1913	A primary gastrinoma in the submucosa of the jejunum. A lymph node metastasis (1.5 cm) in the jejunal mesentery 5 cm distal to the ligament of Treitz	NP	Partial resection of jejunum and pancreatocduodenectomy (on the suspicion of multiple primary sites)	7 months	Alive and remained eugastrinemic
Wu et al. 1997 [3]	M/35	460	Jejunum (distance from the ligament of Treitz not mentioned)	NP	Tumor resection by enucleation	134 months	Elevated FSG at 2 years, local recurrence at 11 years

*M* male, *F* female, *FSG* fasting serum gastrin, *NP* not provided

Gastrinomas of the jejunum are usually small in size, with a reported diameter of 0.5–3 cm [4, 5]. There has only been one case where the tumor was identified preoperatively by combined imaging studies [5]. Preoperative imaging studies have failed to identify a primary tumor in the other reported cases. The unique feature of our case is that the tumor was large in size, and may be the largest tumor reported to date. Preoperative ultrasound and abdominal CT correctly localized the ectopic gastrinoma.

Although gastrinomas tend to be slow growing, 60 to 90 % of them were associated with metastases [8–10]. Like other neuroendocrine tumors, malignancy cannot be established cytologically, but is determined by the presence of metastases as well as vascular or lymphatic invasion [11]. Larger gastrinomas are more likely to metastasize; liver and regional lymph nodes are the most common sites of metastases. Our case is unusual in that the tumor was large in size, yet there is no evidence of distant metastasis.

Many of the gastrinomas are small in size, which makes preoperative localization highly challenging. Furthermore, since the jejunum is not located within the gastrinoma triangle, this unexpected localization makes the diagnosis difficult. A number of tests are used to localize gastrinomas. CT scan, MRI, and ultrasound are used widely as the initial methods for localizing gastrinoma and are excellent for visualization of larger tumors. Somatostatin receptor scintigraphy (SRS) has a high sensitivity for assessing the extent of the disease. In recent years, SRS has become the imaging study of choice for identifying primary tumors and metastatic lesions in ZES [12–15]. However, the sensitivity of SRS to detect gastrinomas is highly dependent on its size, ranging from 30 % for gastrinomas smaller than 1.1 cm to 96 % for gastrinomas larger than 2 cm [15]. EUS has a high sensitivity for detection of pancreatic gastrinomas, ranging from 75 to 100 % [16, 17]. However, EUS is not as accurate in detecting duodenal gastrinomas [18]. These imaging studies should be used in conjunction to achieve optimal preoperative assessment of the gastrinoma. It is important to note that smaller tumors (less than 1 cm) may often be missed and may require the use of IOUS to facilitate localization of small lesions that were not readily visible on preoperative studies. Current transducers can detect lesions as small as 2 mm [19]. In our case, preoperative abdominal CT and EUS and intraoperative US were use for localization of the gastrinoma and rule out metastatic disease. SRS was not performed because it was not available at our institute at the time of diagnosis in 2003.

The discovery of a gastrinoma in the jejunum raises the question of whether the tumor is a primary or metastatic lesion. Exhaustive preoperative imaging as well as careful surgical exploration during laparotomy including intraoperative ultrasound revealed no additional lesions. Thus, the large jejunal tumor was considered to be the primary lesion. Still, it was possible that an occult primary lesion was missed by EUS, surgical exploration, and intraoperative ultrasound. However, plasma gastrin level normalized shortly after the excision of the tumor. Over 10 years after surgery, the patient is still eugastrinemic, and there is no evidence of tumor recurrence in long-term follow-up. Based on these findings, it is apparent that the jejunal tumor is a solitary primary gastrinoma.

The current treatment of sporadic, resectable, non-metastatic gastrinomas is early surgical exploration and surgical resection. Reviews have stated that for sporadic gastrinomas, the initial postoperative cure rate is approximately 50–60 %, and the long-term cure rate is 35–40 % [20–22]. There are a limited number of studies addressing the long-term follow-up of ectopic gastrinomas. Wu el al. treated eight patients with ectopic gastrinomas from 1982 to 1997, seven patients were cured biochemically after resection, and five patients were cured with a mean follow-up of 7.5 years (range, 0.4–11.7 years) [3]. In our case, after surgical excision of the gastrinoma located in the jejunum as the lone method of treatment, the patient was still eugastrinemic and asymptomatic without tumor recurrence at 10-year follow-up. Among the six reported cases of gastrinomas originating from the jejunal wall, only three cases reported a follow-up period of more than 3 years [3, 5, 6]. Antonioli et al. reported a case with gastrinomas in the jejunal wall with metastatic lymph nodes in the adjacent mesentery. After resection of the involved jejunum and mesentery, she remained eugastrinemic 3 years after operation [5]. Katoh et al. reported a case of gastrinoma in the jejunum and jejunal mesentery with multiple liver metastases. Ten years after tumor resection, the patient is alive and there was stabilization of liver metastasis [6]. Wu et al. reported a case of primary jejunal gastrinoma treated by enucleation, who had biochemical recurrence at 2 years after surgery and local recurrence detected by SRS at 11 years after surgery [3].

## Conclusions

Although primary jejunal gastrinomas are rare occurrences, it should be considered during the differential diagnosis of jejunal tumors if classic symptoms, such as uncontrolled reflux, non-healing mucosal ulcers or diarrhea are present. Here, we report an extremely rare case of a primary jejunal gastrinoma in which the tumor may be the largest reported to date. Over 10 years after segmental resection of the jejunum containing the gastrinoma, the patient is still eugastrinemic and asymptomatic with no evidence of tumor recurrence.

## Consent

Written informed consent was obtained from the patient for publication of this Case report and any accompanying images. A copy of the written consent is available for review by the Editor-in-Chief of this journal.

## Abbreviations

ZES: Zollinger-Ellison Syndrome
CT: computed tomography
AFP: alpha-fetoprotein
CEA: carcinoembryonic antigen
CA 19-9: carbohydrate antigen 19-9
EUS: endoscopic ultrasound
US: ultrasonography
MRI: magnetic resonance imaging
SRS: somatostatin receptor scintigraphy
